# Mitochondrial and Autophagy‐Related Proteins are Downregulated in the Brains of the rTg4510 Mouse Model of Tauopathy and Associated with Pain‐like Behavior in a Sex‐Dependent Manner

**DOI:** 10.1002/alz70855_107017

**Published:** 2025-12-25

**Authors:** Larissa J Strath, Airam Vivanco‐Estela, Jinying Yang, Zhiyong Cheng, Zachary Strickland, Robert Caudle, Jada Lewis, Yenisel Cruz‐Almeida

**Affiliations:** ^1^ University of Florida, Gainesville, FL, USA

## Abstract

**Background:**

The relationship between dementias and pain remains under‐researched, despite individuals with chronic pain having increased likelihood of developing dementia. Tauopathies, a factor in the pathology of many dementias is characterized by pathological tau protein aggregation and are associated with mitochondrial dysfunction and impaired autophagy. Moreover, previous literature suggests that changes in mitochondrial complexes in humans are associated with increased pain sensitivity and chronic pain development. In this experiment, we sought to investigate the relationship between mitochondrial complexes and key autophagy‐related proteins with pain‐like behavior in the rTg4510 tauopathy mouse model.

**Methods:**

Equal number of male and female wildtype mice (*n* = 27), 2‐times tau_P301L_ expression mice (2xTau Tg4510; *n* = 18) and 13‐times tau_P301L_ expression mice (13xTau rTg4510; *n* = 17) aged to ∼11 months underwent reflexive (Von Frey and Hargreaves), and non‐reflexive (orofacial pain assessment device (OPAD)) pain testing. Mice were euthanized and brains were extracted and flash frozen. Western blot analyses quantified protein expression levels of mitochondrial complexes I‐V, mitochondrial chaperone proteins heat shock protein 60 (Hsp60) and 90 (Hsp90), and autophagy facilitating proteins Microtubule‐associated protein 1A/1B‐light chain 3 (LC3) and Beclin‐1. One‐way analyses of variance (ANOVA) tested group differences while correlation analyses tested behavior‐protein associations.

**Results:**

Compared to wildtype mice, the 2xTau Tg4510 mice had more pronounced downregulation of mitochondrial CII, autophagy facilitating proteins, and mitochondrial chaperone proteins while the 13xTau rTg4510 brains did not. Overall, the 2xTau Tg4510 mice demonstrated more sensitivity across multiple reflexive and operant pain‐like behavior assays (*p*'s <0.05). The mitochondrial complexes, autophagy and chaperone protein expression levels were significantly correlated with pain‐like behaviors, primarily in the 2xTau Tg4510 group, in a sex‐dependent manner (*p*'s <0.05).

**Conclusions:**

The findings in the rTg4510 model are considered relevant to dementias, as tau pathology and mitochondrial dysfunction are key features of the diseases. Currently, the rTg4510 model showed significant mitochondrial and autophagy‐related protein downregulation, indicative of aberrant functioning in these pathways. These differences were related to pain‐like behavior in the 2xTau Tg4510 group. Our findings provide insight into the relationship between pain and mitochondrial and autophagy dysregulation in tauopathies, especially in the earlier stages.

